# Dissecting the Supramolecular Dispersion of Fullerenes by Proteins/Peptides: Amino Acid Ranking and Driving Forces for Binding to C_60_

**DOI:** 10.3390/ijms222111567

**Published:** 2021-10-26

**Authors:** Tainah Dorina Marforio, Alessandro Calza, Edoardo Jun Mattioli, Francesco Zerbetto, Matteo Calvaresi

**Affiliations:** Dipartimento di Chimica “Giacomo Ciamician”, Alma Mater Studiorum—Università di Bologna, 40126 Bologna, Italy; alessandro.calza2@studio.unibo.it (A.C.); edoardojun.mattioli2@unibo.it (E.J.M.); francesco.zerbetto@unibo.it (F.Z.)

**Keywords:** fullerene, amino acids, proteins, peptides, molecular dynamics simulations, nanobiotechnology, nanobio interface, MM/GBSA, π–π stacking

## Abstract

Molecular dynamics simulations were used to quantitatively investigate the interactions between the twenty proteinogenic amino acids and C_60_. The conserved amino acid backbone gave a constant energetic interaction ~5.4 kcal mol^−1^, while the contribution to the binding due to the amino acid side chains was found to be up to ~5 kcal mol^−1^ for tryptophan but lower, to a point where it was slightly destabilizing, for glutamic acid. The effects of the interplay between van der Waals, hydrophobic, and polar solvation interactions on the various aspects of the binding of the amino acids, which were grouped as aromatic, charged, polar and hydrophobic, are discussed. Although π–π interactions were dominant, surfactant-like and hydrophobic effects were also observed. In the molecular dynamics simulations, the interacting residues displayed a tendency to visit configurations (i.e., regions of the Ramachandran plot) that were absent when C_60_ was not present. The amino acid backbone assumed a “tepee-like” geometrical structure to maximize interactions with the fullerene cage. Well-defined conformations of the most interactive amino acids (Trp, Arg, Met) side chains were identified upon C_60_ binding.

## 1. Introduction

In biomedical applications, fullerenes have wide potential [1,2,3] due to their (i) antioxidant and radical scavenging capacity; (ii) neuroprotective action; iii) biological activity as antiviral, antibacterial, antiapoptotic molecules; (iv) enzyme inhibition activity; (v) photosensitizing ability in photodynamic anticancer and antimicrobic therapy; (vi) capacity as contrast agents; and (vii) drug and gene delivery ability. The extreme hydrophobic nature of fullerenes [4] has, however, prevented their biological application, making them of scarce bioavailability in physiological environments. Different strategies have been explored to promote fullerenes’ dispersion in water. Initial studies of the biological activity of fullerenes involved the functionalization of the hydrophobic cage with hydrophilic moieties to enhance their water solubility [5,6]. For instance, carboxy- and malonyl-derivatives of C_60_ were demonstrated to be an interesting class of reactive oxygen species( ROS) scavenging agents [7,8,9,10]. However, the most appealing fullerene derivatives for biological applications are fullerene–biomolecule conjugates [11], such as fulleroamino acids and fulleropeptides [12,13,14,15], in which the functionalization moiety is both hydrophilic and biocompatible. Fulleroamino acids and fulleropeptides are at least partially water soluble, and demonstrated high biological and pharmacological activities [12,13,14,15]. Fullerenes were also chemically conjugated to proteins [15,16,17] such as azurin [16], thyroglobulin [17], and albumins [17] to improve their functionality (electronic communication or targeting) and biocompatibility.

The direct derivatization of a fullerene with amino acids or its chemical conjugation with peptides and proteins makes a fullerene biocompatible and improves its water solubility, but, as with any fullerene functionalization procedure, these processes degrade the peculiar chemical-physical properties of C_60_ [18]. Supramolecular approaches can preserve these properties [19]. Recently, it was demonstrated that it is possible to use peptides [20] and proteins [21,22,23,24,25,26,27,28,29] as supramolecular hosts for the non-covalent dispersion of fullerenes [21]. Different proteins, such as albumins [22,23], lysozyme [23,24,25,26], pepsin [27], trypsin [27], or natural protein surfactants [28] were used for the mono-molecular dispersion of pristine fullerenes in a physiological environment.

Pioneering approaches based on reverse docking were attempted to identify proteins characterized by one or more hydrophobic pockets suitable for accommodating a fullerene [30,31,32]. Proteins were also engineered into scaffolds for C_60_ binding on the protein surface [33,34].

However, a full comprehension of the process governing the association of peptides/proteins with fullerenes is still lacking. Detailed knowledge about the formation of complexes of peptides/proteins with C_60_ can advance bio-based applications of fullerenes [35]. For instance, inhibitors can be developed by increasing the peptide/fullerene binding [32,36,37,38], the antioxidant properties of fullerenes can be enhanced by protein interaction [39], and fullerenes’ ability to generate ROS under irradiation can be tuned [20,23,25,26,40].

Amino acids are the elementary building blocks of proteins and, therefore, they must be the starting point for investigating the complex phenomena that govern the interactions between peptides/proteins and fullerenes [41,42].

In this study, we determined the adsorption energies of all twenty natural amino acids with C_60_, ranked their propensity to interact with C_60_, and identified the thermodynamic contributions responsible for their interactions. The atomistic details of the interaction between amino acids and fullerene were obtained using molecular dynamics (MD) simulations. MD simulations are a powerful tool to unravel the mechanisms of interaction of proteins with carbon nanomaterials, providing detailed structural information [43]. An analysis of the binding components of the energy was performed by using Molecular Mechanics/Generalized Born Surface Area (MM/GBSA) and Molecular Mechanics/Poisson–Boltzmann Surface Area (MM/PBSA) methodologies [44]. The analysis of the energy contributions to the binding between amino acids and C_60_ can offer guidelines of general applicability to understand and design protein–fullerene interactions. This information is crucial to (i) understand in detail the driving forces in the interaction between peptides/proteins and fullerenes, (ii) enhance our ability to engineer such complexes, (iii) predict a priori the strength of adsorption and structure of a protein upon fullerene binding.

## 2. Results and Discussion

**Scheme 1:**
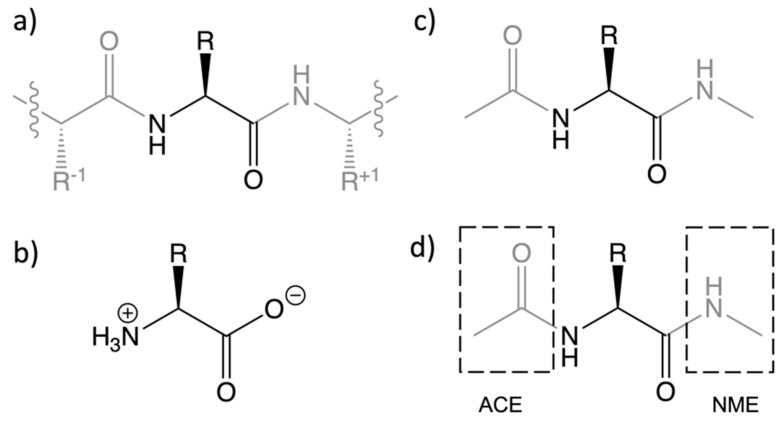
(**a**) Amino acid (in black) inserted in a peptide/protein sequence. (**b**) Amino acid in zwitterionic form. (**c**,**d**) Amino acid capped with ACE/NME.

The geometries of the twenty C_60_-amino acid complexes were built. The amino acids were capped by acetyl (ACE) and n-methyl amide (NME) groups at the N- and C-termini (ACE-AA-NME), (Scheme 1) to reproduce the typical interactions between C_60_ and an amino acid inserted into the peptide/protein sequence.

Subsequently, 100 ns of molecular dynamics simulations were carried out for each complex to sample the possible interaction geometries between the C_60_ and the twenty amino acids. Post-processing of the trajectories allowed us to estimate the binding energy between the amino acids and the C_60_ and the binding components of the energy by MM/GBSA and MM/PBSA.

### 2.1. Ranking the Binding Energies

The interaction energies between the capped amino acids and the C_60_ were calculated at MM/GBSA and MM/PBSA levels of theory (Figure S1). The results of the two methodologies were very similar (MM/GBSA systematically provided a more stabilizing interaction energy of −0.5 kcal mol^−1^ compared to MM/PBSA, due to the different treatment of solvation). In the following, we describe in detail the MM/GBSA ranking, because such calculations are faster (a crucial issue for applications in virtual screening or MD simulations of large biosystems) and mostly because the GB terms are pair-wise decomposable (PB non-polar solvation energies are currently not decomposable) [44].

**Figure 1:**
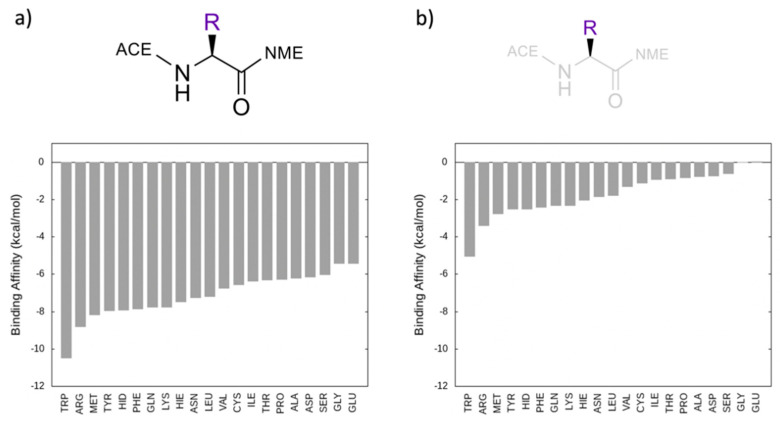
Binding energy between the (**a**) whole capped amino acid and (**b**) amino acid side chain and C_60_ calculated by MM/GBSA for the twenty proteinogenic amino acids.

The ranking obtained by the binding energies of the capped amino acids (Figure 1a) and of the amino acid side chains (Figure 1b) was the same. This implied a constant contribution from the conserved amino acid backbone (with a value of $E_{binding}$ ~ 5.4 kcal mol^−1^, similar to the interaction of Gly with C_60_). The contribution due to the amino acid side chains varied and was responsible for the different interactions of the twenty amino acids (from $E_{binding}=$ −5.0 kcal mol^−1^ of Trp to $E_{binding}=$ +0.1 kcal mol^−1^ of Glu).

**Figure 2:**
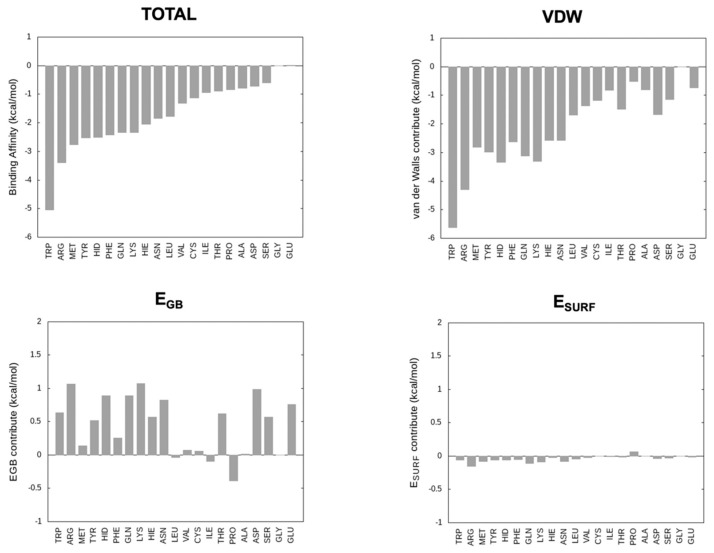
Analysis of the components of the binding energy of the amino acid side chains by MM/GBSA.

Van der Waals interactions are the driving force in binding (Figure 2) [35,45]. It is important to emphasize that in the present model, the van der Waals term includes π–π interactions.

Hydrophobic interactions, i.e., nonpolar solvation (E_SURF_), assist binding, even if the corresponding values are small. Contributions to the nonpolar solvation originate in the hydrophobic parts of some amino acids, such as aliphatic chains or aromatic rings, which, upon the formation of the complex with C_60_, come into contact with the hydrophobic surface of the C_60_ cage instead of water molecules, which interact unfavorably with these regions.

Polar solvation terms (E_GB_) are quite important and deserve an accurate analysis. These terms are detrimental to binding and their contribution generally features an energy level greater than zero, i.e., it is destabilizing. The hydrophilic part of many amino acids, upon interaction with C_60_, is forcedly desolvated, increasing the energy of the system. This is particularly evident in the cases of charged (Arg, Lys, Glu, Asp) and polar (Gln, Asn, Thr, Ser) amino acids: these residues, after interaction with C_60_, are screened from water and are no longer able to maintain a full solvation shell. Other amino acids commonly considered hydrophobic, such as Trp, Tyr, and His, are also characterized by positive desolvation energy, albeit to a lesser extent. This behavior is due to the presence of hydrophilic moieties, such as the N-H or O-H groups, which are desolvated upon C_60_ binding. Of course, purely hydrophobic residues, such as Met, Leu, Val, Ile, Pro, Ala and Gly, do not suffer this energy penalty upon binding.

#### 2.1.1. Aromatic Amino Acids

**Figure 3:**
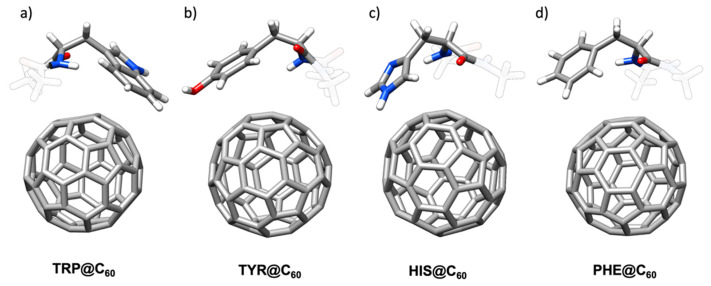
Snapshots from the MD of the interactions of aromatic amino acids (**a**) Trp, (**b**) Tyr, (**c**) His and (**d**) Phe with C_60_. For each amino acid, the most interactive configuration is shown.

If we consider the extended π-system of the fullerene, it is not surprising that aromatic amino acids such as tryptophan, tyrosine, phenylalanine, and histidine interacted the most with it (Figure 3) [20,24,33,34,35,39,46,47,48,49,50,51,52,53,54]. In particular, Trp exhibited the largest value of binding energy among the twenty proteinogenic amino acids. Trp is commonly recognized as the amino acid that most interacts with carbon nanoparticles, such as carbon nanotubes [55] or graphene [56]. The large indole group of tryptophan also demonstrated the largest interaction with the C_60_ ($E_{binding}=$ −5.0 kcal mol^−1^), followed by tyrosine ($E_{binding}=$ −2.5 kcal mol^−1^), histidine ($E_{binding}=$ −2.5 kcal mol^−1^), and phenylalanine ($E_{binding}=$ −2.4 kcal mol^−1^).

Experimentally, in C_60_@lysozyme, π-stacking interactions between C_60_ and Trp (sandwich-like for Trp62 and T-shape-like for Trp63) are crucial for binding [24,35,51]. Analogously, in a peptide designed to recognize C_60_, C_60_ is wedged between two Tyr residues [33]. Moreover, consensus tetratricopeptide repeat proteins (CTPR) were identified as good candidates for hosting fullerenes due to the high abundance of tyrosine (six residues per repeat unit) [34]. Finally, phenylalanines were used as recognizing moieties in peptidic nanotweezers [20].

#### 2.1.2. Charged Amino Acids

**Figure 4:**
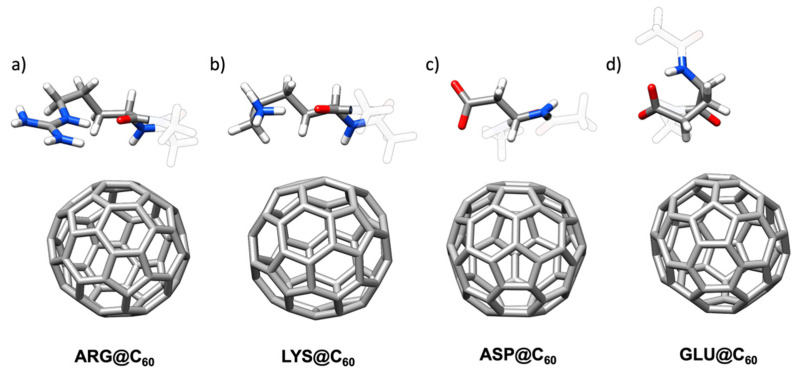
Snapshots from the MD of the interactions of charged amino acids (**a**) Arg, (**b**) Lys, (**c**) Asp and (**d**) Glu with C_60_. For each amino acid, the most interactive configuration is shown.

Besides π–π interactions, electron rich fullerenes can also interact with amino acids through (i) cation-π [54], and (ii) non-standard hydrogen bonds, in which C_60_ acts as a hydrogen bond acceptor [47]. For this reason, positively charged amino acids, such as arginine ($E_{binding}=$ −3.4 kcal mol^−1^) or lysine ($E_{binding}=$ −2.3 kcal mol^−1^), interacted strongly with the C_60_, while negatively charged amino acids, such as aspartate ($E_{binding}=$ −0.7 kcal mol^−1^) and glutamate ($E_{binding}=$ +0.1 kcal mol^−1^) were among the least interacting residues (Figure 4).

After Trp, Arg was the second amino acid that most strongly interacted with the C_60_. The high affinity of Arg with carbon nanomaterials is well-known experimentally [57,58] and widely discussed computationally [57,59,60,61]. The affinity of Lys with C_60_ is lower than that of Arg, although both are positively charged and their side chains are characterized by the same surface (the accessible surface area, ASA, is 2.8 nm^2^ for Lys and 2.9 nm^2^ for Arg). The presence of the guanidinium group plays a crucial role in the preferential adsorption of Arg onto carbon nanomaterials because of the presence of: (i) the π–π interactions that the planar guanidinium group establishes with C_60_ (the vdW interactions of the Arg side chain are 4.3 kcal mol^−1^, while for Lys they are 3.3 kcal mol^−1^); (ii) the surfactant-like behavior of Arg when interacting with hydrophobic nanoparticles. The Arg residues orient themselves so that the guanidinium head anchors on the cage of the fullerene, while the aliphatic chain sticks to its hydrophobic surface. In this way, Arg effectively becomes a surfactant that creates an amphiphilic shield around the hydrophobic nanoparticle [61].

#### 2.1.3. Polar Amino Acids

**Figure 5:**
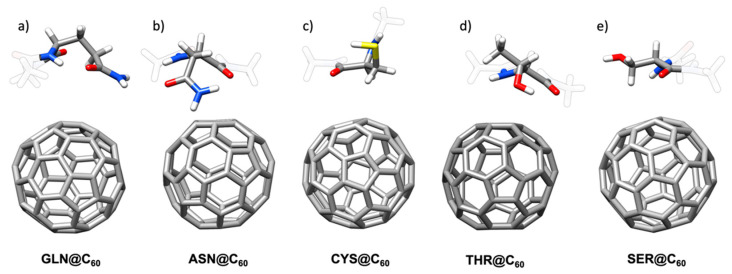
Snapshots from the MD of the interactions of polar amino acids (**a**) Gln, (**b**) Asn, (**c**) Cys, (**d**) Thr and (**e**) Ser with C_60_. For each amino acid, the most interactive configuration is shown.

Polar amino acids, such as glutamine, asparagine, cysteine, threonine, and serine, are amphiphilic residues that interact with C_60_, producing surfactant-like interactions (Figure 5).

In this case, the hydrophobic part of the residues interacted with the fullerene surface, while the hydrophilic part continued to interact with water. The affinity of the residues is directly proportional to the vdW interactions that the hydrophobic chain established with the C_60_ cage. For example, Gln ($E_{binding}=$ −3.1 kcal mol^−1^) interacted more strongly than Asn ($E_{binding}=$ −2.6 kcal mol^−1^) due to a longer aliphatic chain, and Thr ($E_{binding}=$ −1.5 kcal mol^−1^) interacted more strongly than Ser ($E_{binding}=$ −1.1 kcal mol^−1^) due to the presence of an additional methyl group. The affinity of the residues was also inversely proportional to the desolvation energy penalty. Cys interacted more strongly than Ser because the desolvation of the more polar -OH group (0.6 kcal mol^−1^) represented a higher energy penalty than the desolvation of the less polar -SH group (0.1 kcal mol^−1^).

#### 2.1.4. Hydrophobic Amino Acids

**Figure 6:**
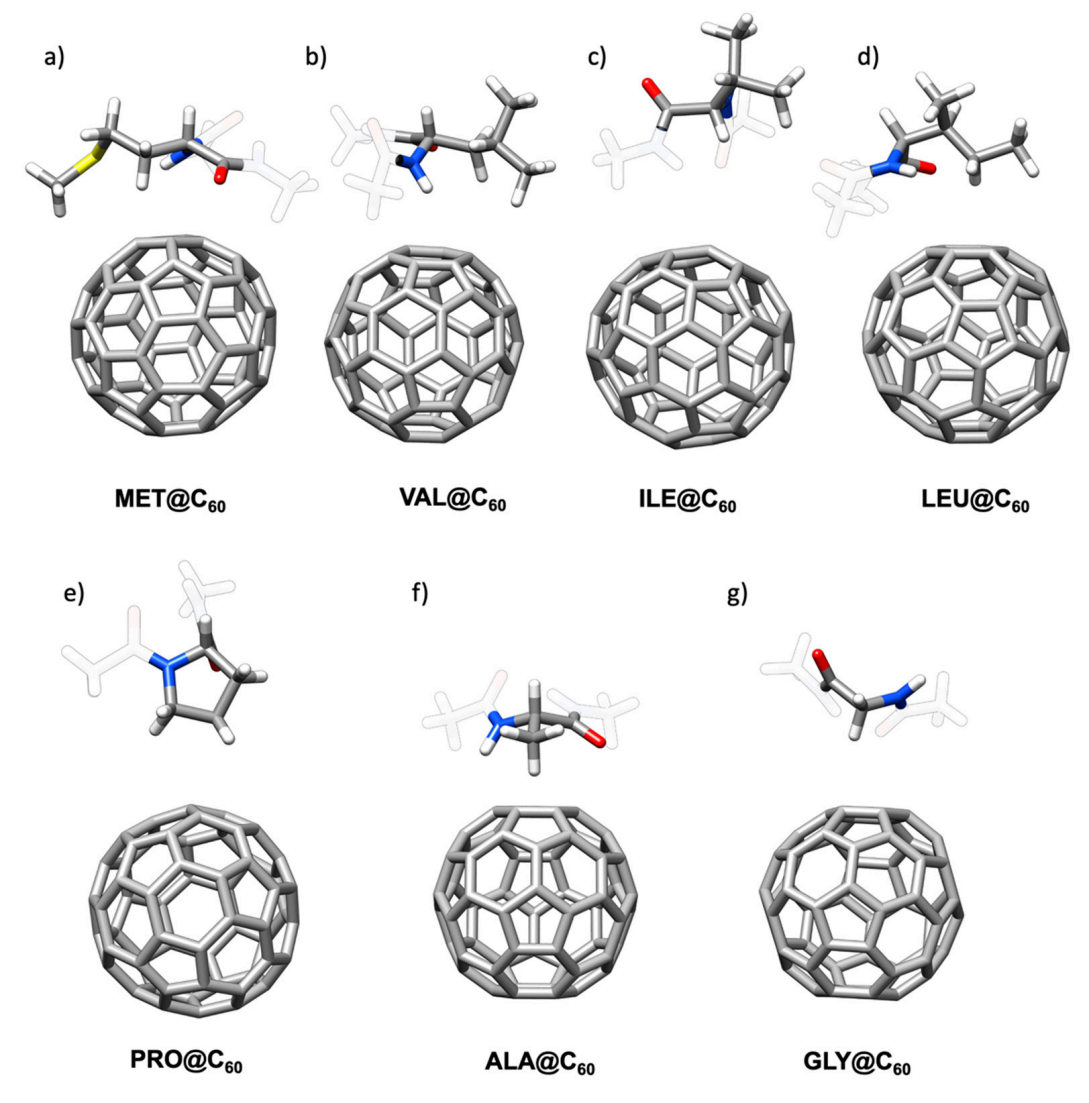
Snapshots from the MD of the interactions of hydrophobic amino acids (**a**) Met, (**b**) Val, (**c**) Ile, (**d**) Leu, (**e**) Pro, (**f**) Ala and (**g**) Gly with C_60_. For each amino acid, the most interactive configuration is shown.

In protein/peptide-fullerene complexes, several aliphatic residues are usually part of the C_60_ binding pocket and contribute to binding [33,35,49]. Hydrophobic interactions can be established with methionine, valine, isoleucine, leucine, proline, alanine, and glycine (Figure 6). The hydrophobic effect can be defined as the absence of a desolvation penalty in the binding of a residue with the C_60_; indeed, even negative values in the E_GB_ are found. Concurrently, the larger the contact area between the amino acid side chain and the C_60_, the larger the stabilizing effect due to vdW interactions (dispersion forces). Shape complementarity represents a quick way to estimate both the stabilizing van der Waals and hydrophobic interactions between proteins and carbon nanomaterials [21,45,62,63].

In this context, the behavior of Met deserves special attention. Met was the amino acid with the third strongest level of interaction in the ranking in this study. The presence of a sulfur atom strongly favors the interaction of the side chain with the C_60_. This stabilizing interaction can be interpreted as the soft sulfur atom that strongly interacts with the soft and electron-rich fullerene cage. In practice, a deep potential energy well is formed by the interaction of the S atom of methionine with the C atoms of the C_60_. Such strong stabilization is not present for pure aliphatic chains such as Val, Ile, Leu.

### 2.2. Conformations of Amino Acids in Water and Upon Interaction with C_60_

To investigate the effect of the absorption of amino acids on C_60_, for the three most interactive residues (Trp, Arg, and Met) we carried out a thorough conformational analysis of the amino acids in water and upon C_60_ binding.

#### 2.2.1. Analysis of the Torsional Angles of the Amino Acid Backbone

**Figure 7:**
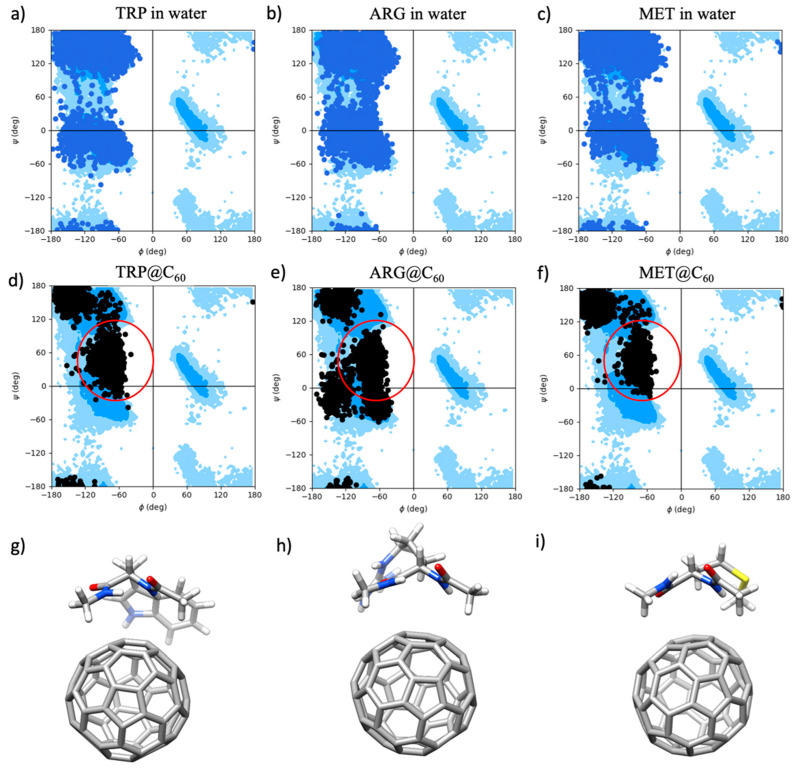
The distribution of the backbone torsional angles (**a**–**f**) obtained for Trp, Arg, and Met in (**a**–**c**) bulk water and (**d**–**f**) upon C_60_ binding. The background regions (shown in cyan) represent the sterically allowed Ramachandran angles. Blue (**a**–**c**) and black (**d**–**f**) points represent the torsional angles phi (φ) and psi (ψ) of the amino acids observed during the MD simulations. (**g**–**i**) Representative structures of the “tepee-like” conformations of Trp@C_60_ (**g**), Arg@C_60_ (**h**), and Met@C_60_ (**i**).

We sampled the torsional angles phi (φ) and psi (ψ) of the amino acid backbone during the MD simulations in water and upon interaction with C_60_. The simulations of the amino acids in water showed that the “classic” and more stable regions of the right-handed α-helix and β-sheet were the most populated (Figure 7a–c). By contrast, the binding with C_60_ altered the φ-ψ distributions by affecting the conformations observed in water and the amino acids sampled a new φ-ψ region (indicated by a red circle in Figure 7d–f), which was rarely observed in bulk water. The amino acids in this region of the Ramachandran plot assumed a “tepee-like” geometrical structure to optimize the interactions with the C_60_ cage of the side chain of the amino acid and the N-terminal and C-terminal peptide bonds, as represented in Figure 7g–i.

#### 2.2.2. Analysis of the Side Chain Conformation of the Amino Acids upon C_60_ Binding

**Figure 8:**
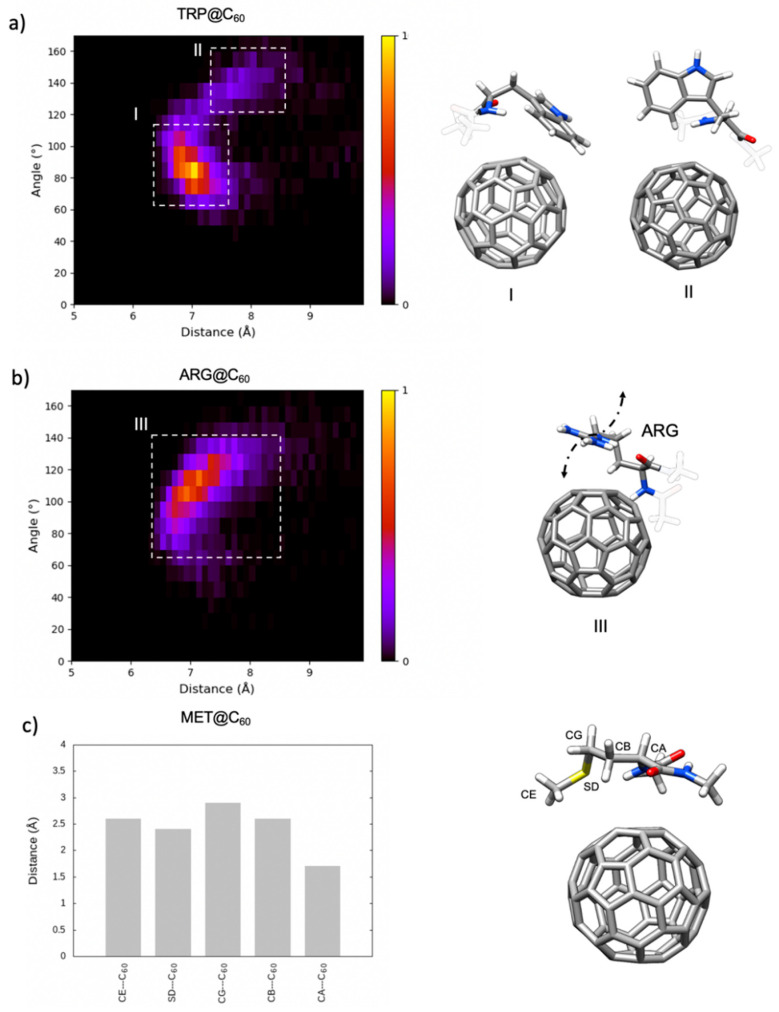
(**a**,**b**) Normalized free energy surfaces of (**a**) Trp@C_60_ and (**b**) Arg@C_60_. In (**a**) the x axis represents the distance between the center of mass (COM) of the indole and the COM of the C_60_. The y axis represents the angle between the (i) COM(C_60_)—COM (indole) and (ii) indole N-H vectors. In (**b**) the x axis represents the distance between the center of mass (COM) of the guanidinium group and the COM of the C_60_. The y axis represents the angle between the (i) COM(C_60_)—COM (guanidinium) and (ii) guanidinium CZ-NH1 vectors. (**c**) Mean distance during the MD simulation between COM (C_60_) and CA, CB, CG, SD, and CE atoms in Met.

Orientational preferences of the side chains of the amino acids upon C_60_ binding clearly appeared in the Free Energy Surfaces, FES, of the complexes of Trp and Arg (Figure 8 and Figure S2)_._

The FES of Trp@C_60_ displayed the existence of two minima. In the more populated/stable minimum, the conformation was sandwich-like. It was characterized by π–π interactions between the indole ring of Trp and the fullerene cage (conformation I in Figure 8a). In the less populated minimum, the conformation was T-shape-like; the indole group assumed a perpendicular configuration with respect to the fullerene cage (conformation II in Figure 8a). These conformations were experimentally observed and discussed in the C_60_@lysozyme complex, in which sandwich-like interactions were recognized for Trp62 and T-shape-like interactions for Trp63.

The FES of Arg@C_60_ displayed a single minimum, corresponding to the establishment of π–π interactions between the planar guanidinium group and the C_60_. This minimum was broader if compared with that of the Trp complex because the smaller guanidinium group adhered less to C_60_, if compared to the indole group, and may have rotated freely towards the C_60_ surface.

In the case of Met@C_60_ we analyzed the gluing of the Met side chain to the C_60_ cage. The CA atom was anchored to C_60_ by the interactions of the amino acid also with its backbone, and the mean distance during the MD simulation was small (1.7 Å). However, all the other atoms of the aliphatic side chain remained attached to the surface of the C_60_ during the MD simulation in a “hug” to the fullerene cage. The mean distances of all the atoms of the Met side chain remain close to the C_60_’s surface, (ranging from 2.4 to 2.9 Å) and, unsurprisingly, the sulfur atom acted as the ligand. In fact, even if it was characterized by a larger vdW radius compared to C atoms, the S atom was, on average, the closest to the C_60_ after CA (2.4 Å).

## 3. Materials and Methods

### 3.1. System Setup

Amber force fields ff14SB [64] was used to model the amino acids (AA). The twenty proteinogenic amino acids (Ala, Arg, Asn, Asp, Cys, Glu, Gln, Gly, Hie, Ile, Leu, Lys, Met, Phe, Pro, Ser, Thr, Trp, Tyr, and Val) were considered in their physiological protonation state. For the histidine, both the protomers HID (Nδ1-protonated histidine) and HIE (Nε1-protonated histidine) were taken in account. The amino acids were capped by acetyl (ACE) and n-methyl amide (NMA) groups at the N- and C-termini (ACE-AA-NME), to reproduce the typical interactions between C_60_ and a peptide/protein host. The C_60_ carbon atoms were modelled as uncharged Lennard-Jones particles by using sp2 carbon parameters taken from the ff14SB force field [64].

### 3.2. MD Simulations

AMBER 16 was used to run the simulations [65]. A total of 1000 steps of steepest descent minimization were performed with SANDER on the generated ACE-AA-NME@C_60_ complexes. The minimized structures (which were only cleared from severe steric clashes) were considered for a 1 ns equilibration step, and heated from 0 to 300 K (Langevin thermostat). Periodic boundary conditions (PBC) and Particle Mesh Ewald summation were used throughout (with a cut-off radius of 10 Å for the direct space sum). The MD simulations of the amino acids were performed with explicit solvent by using the TIP3P water model. Chloride or sodium counterions were included to exactly neutralize the charged amino acids. At the end of the equilibration process, a production MD of 100 ns was carried out for every system at 300 K.

### 3.3. Molecular Mechanics/Generalized Born Surface Area (MM/GBSA) and Molecular Mechanics/Poisson–Boltzmann Surface Area (MM/PBSA) Analysis

A total of 4000 snapshots were used for the MM/GB(PB)SA analysis [44]. An infinite cut-off was used for all the interactions. The electrostatic contribution to the solvation free energy was calculated with the Poisson–Boltzmann (PB) model or the Generalized Born (GB) model, as implemented in MMPBSA.py [66]. The nonpolar contribution to the solvation free energy was determined with solvent-accessible surface-area dependent terms.

### 3.4. Conformational Analysis of the Amino Acids

The Ramachandran plots and FES were obtained using MDAnalysis software [63,64]. The analysis of distances and angles in the MD trajectories was carried out by CPPTRAJ [67], as implemented in AMBER 16. The energy landscape of the amino acids interacting with C_60_ was visualized in terms of free-energy, which was projected as contour lines onto a two-dimensional space formed by two internal coordinates of the system. The free-energy change associated to the passage between two different states was ΔG = −RT (ln p_1_/p_2_), where R is the ideal gas constant, T is the temperature, and p_i_ is the probability of finding the system in state i. The two-dimensional space defined by the selected coordinates was divided into a grid and the free energy calculated for each bin of the grid. The p_i_ values correspond to the number of times the molecular dynamics trajectory “visits” a given bin. The whole set of G values was normalized [68,69] in such a way that the lowest value (i.e., the most populated bin) corresponds to 1.

## 4. Conclusions

The potential of fullerenes in bio-medical applications is significant and can only partly be exploited because their extremely hydrophobic nature makes them rare in physiological environments. Of all the strategies that have been developed to make fullerenes bio-available, the “Trojan horse” approach, in which they are stashed away in water-soluble proteins, appears to be the greenest and most environmentally friendly [21]. Propensity rules able to identify where a fullerene can be accommodated in a protein were developed. Molecular dynamics simulations that properly include van der Waals (including π-stacking), hydrophobic, and solvation interactions, were used to determine quantitatively the binding affinity of all the proteinogenic amino acids with C_60_. Van der Waals interactions are the driving forces in binding. Hydrophobic interactions, such as nonpolar solvation, assist in binding, even if the corresponding values are small. Polar solvation terms are detrimental to binding in the cases of charged and polar amino acids. Only purely hydrophobic residues do not suffer this energy penalty upon binding. The complex interplay between these terms makes tryptophan, arginine, and methionine the residues with the strongest interactions. A thorough conformational analysis of these amino acids demonstrated that upon C_60_ binding, the interacting residues show a tendency to visit configurations not “experienced” in water, and the backbone assumes a “tepee-like structure” to maximize the interaction with the C_60_ cage. The binding of Trp is characterized by π–π interactions between the indole ring of Trp and the fullerene cage. These interactions may be sandwich-like (the most common) or T-shaped. Interactions of the π–π type also govern the binding between Arg and C_60_ involving the planar guanidinium group and C_60_. The hydrophobic Met interacts with C_60_ in a “hug” between its aliphatic side chain and the fullerene cage. The presence of a sulfur atom strongly favors the interaction of the aliphatic side chain with C_60_.

## Data Availability

All data in this study can be requested from the corresponding authors (tainah.marforio2@unibo.it, T.D.M.; matteo.calvaresi3@unibo.it, M.C.).

## Supplementary Materials

The following are available online at www.mdpi.com/1422-0067/222/11/1567/s1.
