# Supplementary material for: Dissecting the Supramolecular Dispersion of Fullerenes by Proteins/Peptides: Amino Acid Ranking and Driving Forces for Binding to C60
Source: Int J Mol Sci. 2021 Oct 26;22(21):11567. doi: 10.3390/ijms222111567 (PMC8583719; doi:10.3390/ijms222111567)
Supplement: Supplementary file 1 [file ijms-22-11567-s001.zip › ijms-1434706-supplementary.pdf]

International Journal of *Molecular Sciences*

MDPI

Dissecting the Supramolecular Dispersion of Fullerenes by Proteins/Peptides: Amino Acid Ranking and Driving Forces for Binding to C_{60}

Tainah Dorina Marforio[1,*], Alessandro Calza[1], Edoardo Jun Mattioli[1], Francesco Zerbetto[1] and Matteo Calvaresi[1,*]

[1] Dipartimento di Chimica "Giacomo Ciamician", Alma Mater Studiorum - Università di Bologna, Bologna 40126, Italy

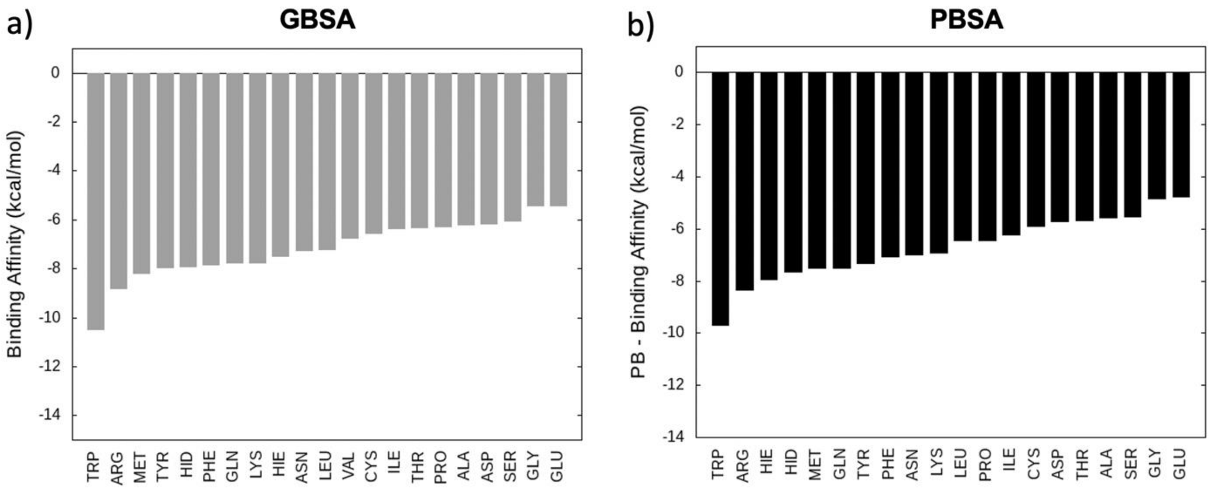

Figure S1. Binding energy between amino acid and C_{60} calculated by a) MM/GBSA and b) MM/PBSA for the twenty proteinogenic amino acids.

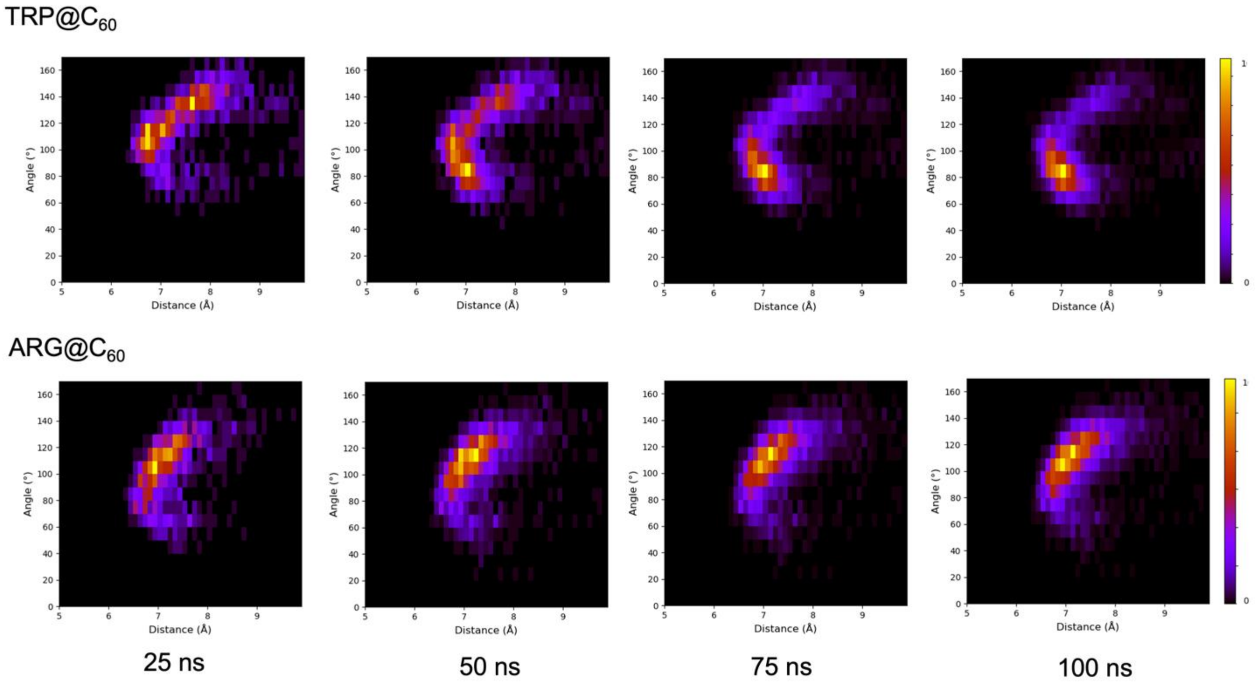

Figure S2. Convergence of the Trp@C_{60} (top) and Arg@C_{60} (bottom) FES as a function of the sampling time.
